# Imaging Diffractometric Biosensors for Label-Free, Multi-Molecular Interaction Analysis

**DOI:** 10.3390/bios14080398

**Published:** 2024-08-17

**Authors:** Cornelia Reuter, Walter Hauswald, Sindy Burgold-Voigt, Uwe Hübner, Ralf Ehricht, Karina Weber, Juergen Popp

**Affiliations:** 1 Leibniz Institute of Photonic Technology, Member of Leibniz Health Technologies, Member of the Leibniz Centre for Photonics in Infection Research (LPI), 07745 Jena, Germany; sindy.burgold-voigt@leibniz-ipht.de (S.B.-V.); uwe.huebner@leibniz-ipht.de (U.H.); ralf.ehricht@leibniz-ipht.de (R.E.); karina.weber@leibniz-ipht.de (K.W.); juergen.popp@leibniz-ipht.de (J.P.); 2 Institute of Physical Chemistry and Abbe Center of Photonics, Friedrich Schiller University Jena, 07743 Jena, Germany; 3 InfectoGnostics Research Campus Jena, Center for Applied Research, 07743 Jena, Germany; 4 Cluster of Excellence Balance of the Microverse, Friedrich Schiller University Jena, 07743 Jena, Germany

**Keywords:** optical biosensors, diffractometric imaging, diffractive biosensor, nucleic acid detection, protein detection, label-free interaction analysis

## Abstract

Biosensors are used for the specific and sensitive detection of biomolecules. In conventional approaches, the suspected target molecules are bound to selected capture molecules and successful binding is indicated by additional labelling to enable optical readout. This labelling requires additional processing steps tailored to the application. While numerous label-free interaction assays exist, they often compromise on detection characteristics. In this context, we introduce a novel diffractometric biosensor, comprising a diffractive biosensor chip and an associated optical reader assembly. This innovative system can capture an entire assay, detecting various types of molecules in a label-free manner and present the results within in a single, comprehensive image. The applicability of the biosensor is assessed for the detection of viral DNA as well as proteins directly in human plasma, investigating different antigens. In our experiments, we achieve a detection limit of 4.2 pg/mm², which is comparable to other label-free optical biosensors. The simplicity and robustness of the method make it a compelling option for advancing biosensing technologies. This work contributes to the development of an imaging diffractometric biosensor with the potential for multiple applications in molecular interaction analysis.

## 1. Introduction

Optical biosensors combine high sensitivity and specificity with non-invasive and versatile real-time detection capabilities. Their ability to work in different environments, coupled with the potential for miniaturization and multiplexing, makes them a highly desirable choice for many applications, ensuring fast, reliable, and cost-effective analytical solutions [1,2,3]. These biosensors are based on several principles, each tailored to address specific challenges. Optical biosensors employ capture molecules immobilized on a substrate to facilitate the specific binding of analytes, serving as the biorecognition sensing element. Additionally, they integrate a detection method to capture and interpret binding events, incorporating a transducer for signal output. The classification of optical biosensors generally falls into two modes: label-free and label-based [4,5].

Due to their exceptional sensitivity, dye-based methods such as fluorescence-, absorbance-, or nanoparticle labeling (ELISA [6], lateral flow [7], colorimetric, fluorescence or luminescence methods [8], or LSPR [9]) are commonly employed in diagnostic assays. However, these labeling approaches come with notable drawbacks. First, they require additional processing steps, introducing complexities in terms of time, extra chemicals, and susceptibility to errors. Second, the selected label must be universally suitable for all analytes in an assay and must remain insensitive to impurities. This is particularly challenging in assays involving multiple analytes, such as the simultaneous detection of DNA and proteins, where establishing a standardized labeling step proves to be complicated. The implementation of multiplexed detection systems capable of analyzing many binding interactions simultaneously is required due to the intricate nature of many complex binding events. Third, labeling can potentially alter the binding properties of the analyte, occasionally interfering with the process of capturing molecules and biosensor analysis.

To overcome these limitations, label-free assays that exploit the inherent physical properties of analytes for direct detection have emerged. Optically readable properties of analytes are: wavelength and polarization-dependent absorption and refraction, autofluorescence, phosphorescence, and vibrational spectra. Resonance effects, differential schemes, or strongly localized evanescent fields are often used to selectively increase the sensitivity for the analyte of interest compared to other influences such as solvent and temperature. Various label-free approaches [10,11] are employed in biosensor technology. Diffractometric sensor principles measure property changes of a grating composed of analytes by analyzing diffraction efficiency [12,13,14]. Refractometric sensing based on diffraction gratings measures the change in refractive index surrounding a grating by observing the diffraction angle [15,16]. Surface plasmon resonance (SPR) biosensors [17,18,19,20] and localized SPR [21] detect changes in the refractive index within the ambit of thin metal layers or metal nanoparticles. Ellipsometry measures changes in the state of polarization when electromagnetic radiation is reflected or transmitted by a sample [10,11]. Surface-enhanced Raman scattering (SERS) measures the vibrational spectra of single biomolecules based on the Raman effect [22].

Diffractometric biosensors [13] are based on specific spatial distributions of capture molecules on sensor substrates. As the analytes are forced to bind to the probe molecules, this predetermined specific spatial distribution is also formed in the analyte. In contrast, perturbations such as other sample components, impurities, or inhomogeneities, will appear uniformly and unconstrained over the entire substrate. This specific spatial distribution of the analyte can be read out optically with high sensitivity by means of diffraction effects; additionally, it is a specific feature against perturbations. Uniform, high-frequency, grating-like structures are suitable as spatial distributions since high first-order diffraction angles can be realized and the expected signal-to-noise ratio is particularly high [23].

Despite the intrinsic advantages and the increased sensitivity of diffractometric sensors in recent years, they have not yet prevailed over refractometric and labeling-based sensors. The reasons could be the still rather complex manufacturing processes of the sensor chips and the readout devices.

Here, we present a method for fabricating universal pre-patterned, surface-functionalized quartz chips that can later be tailored for a specific sensor application, along with a simple and robust optical setup for the quantitative imaging readout of entire sensor chips. The universal pre-patterned quartz chips are further prepared by spotting different capture molecules, to specifically detect nucleic acids as well as proteins on a functionalized surface.

We demonstrate the efficacy of our imaging diffractometric biosensor by successfully detecting DNA from the human cytomegalovirus and identifying human antibodies against the nucleocapsid (N) and spike proteins (S) of SARS-CoV-2 in human plasma. To showcase the simultaneous detection capabilities for both nucleic acids and proteins, we present the directed binding of antibodies to oligonucleotides and the hybridization of target DNA within a single assay.

We show the advantages of the reported biosensor as such: the ability for label-free and simultaneous detection of biomolecules; easy preparation of universal pre-patterned, functionalized chips; linear and absolute optical path length differenceresponse; high specificity; ability for live readout; simple and robust reader principle with low requirements on optics and camera.

## 2. Materials and Methods

Our imaging diffractometric biosensor is based on a combination of dark-field illumination, spatial modulation of the analyte and band-limited imaging. It consist of a diffractive biosensor chip prepared with specific capture molecules, and an associated optical reader assembly. The optical reader assembly is specially designed to take dark-field images of the biosensor chip selecting only the first diffractive order of the diffractive biosensor chip. This readout strategy is only possible in conjunction with the spatially pre-patterned chips. It enables an increased signal-to-noise ratio without a label by avoiding shot noise of the zeroth diffraction order and efficient suppression of spatially unstructured impurities on the substrate. The raw images are normalized and further processed to quantitatively measure areal mass density in a location-dependent manner.

### 2.1. Diffractive Biosensor Chips

**Figure 1:**
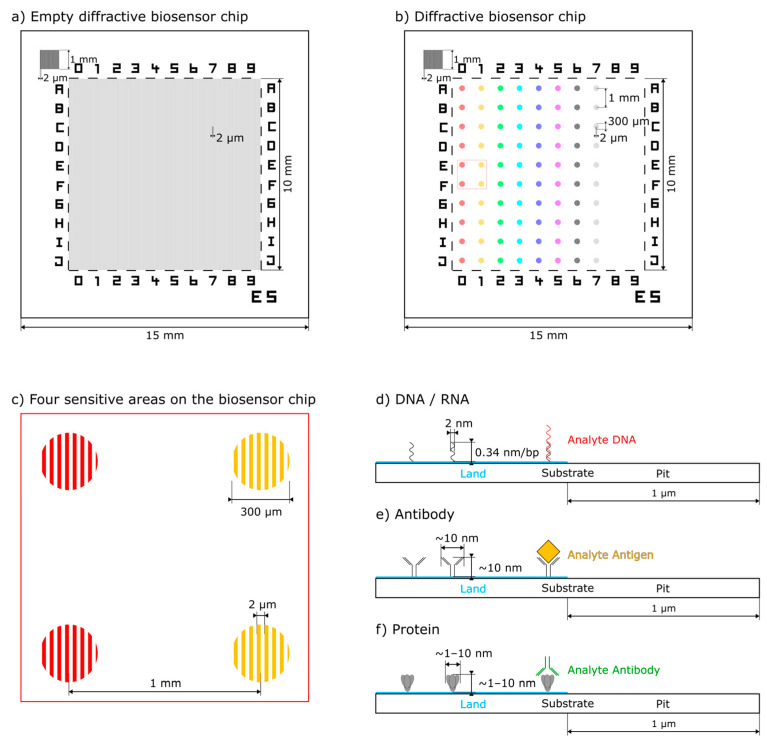
Geometry of the diffractive biosensor chip: (**a**) top view of a diffractive biosensor chip with a strip grating pre-patterned functionalized surface, a numeration matrix, a reference grating (upper left), and a chip number (**b**) prepared chip with 10 spots per column and 8 different conditions per row (indicated by color), (**c**) magnified detail of (**b**) containing 4 strip grating patterned spots with capture molecules, (**d**–**f**) magnified side views of the diffractive biosensor chip with a strip grating pre-patterned functionalized surface (blue) visualizing different application scenarios of (**d**) DNA, (**e**) antigen and (**f**) antibody as analyte, Note: The grating and the biomolecules are not shown to scale.

The sensor principle, presented here, is based on 15 mm × 15 mm biosensor quartz chips with a 10 mm × 10 mm strip grating pre-patterned functionalized surface of 2 µm period (Figure 1a). These chips are further prepared with spots of different capture molecules (Figure 1b). Due to the pre-patterning, the capture molecules are also distributed in a square-wave strip grating with a = 2 µm period and 1:1 duty cycle (1 µm land and 1 µm pit) (Figure 1c). In our work, each column typically consists of 10 spots with d ≈ 300 µm diameter and l = 1 mm distance of the same condition (type of capture molecule and concentration). Each row typically consists of 8 spots of different conditions (Figure 1b). According to the application and the analyte of interest, we show that the diffractive biosensor chip can be prepared for capturing specific DNA, antigens, and antibodies (Figure 1d–f).

We manufacture the diffractive biosensor chip in three steps. First, the numeration matrix, the reference grating, and 61 chips are produced at wafer level on a 600 µm thick 6” quartz wafer by electron beam lithography (Vistec SB350 OS Electron Beam Lithography system with Variable Shaped Beam technology, Vistec Electron Beam GmbH Jena, Germany) and lift-off process of a 100 nm thin gold layer. After this, the wafer is cut into chips to be further processed individually (see Figure 1a but still without light gray grating).

**Figure 2:**
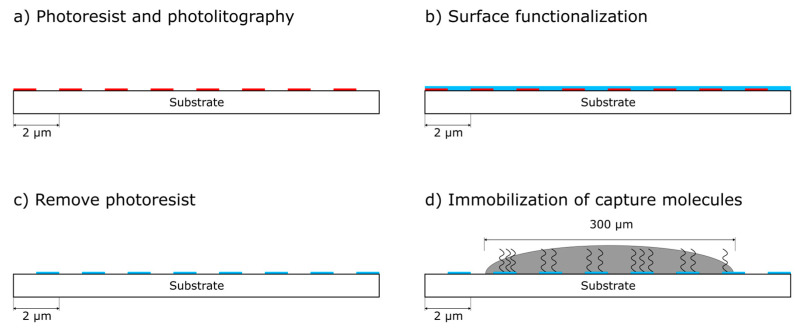
Preparation of the diffractive biosensor chip: (**a**) side view of the diffractive biosensor chip with a photolithographically generated strip grating of photoresist (red), (**b**) surface activation and functionalization (blue), (**c**) resist stripping and (**d**) spotting with binding of specific capture molecules. (not to scale).

Second, the respective specific resist gratings (resist: AZ1505, 600 nm thick) are individually generated photolithographically (clean room of Leibniz IPHT, Jena, Germany) on these prefabricated chips (Figure 1a and Figure 2a). After O-plasma activation of the substrate surface, we have implemented a functionalization procedure using (3-Glycidyloxypropyl)trimethoxysilane (GOPS) [24,25], (Sigma-Aldrich, Steinheim, Germany) deposited in dry toluene (Toluol ROTIDRY, Carl Roth GmbH, Karlsruhe, Germany) in liquid phase Figure 2b [26]. The chemical modification with GOPS is performed for 2 h at 70 °C in 10 mmol/L GOPS in dry toluene under continual mixing. Then the chips are washed with dry toluene and finally, they are dried [27,28]. The procedure is completed by resist stripping via acetone (Figure 2c). The chips are now prepared and can be stored in a vacuum desiccator until they are tailored to a specific application by spotting.

As a third and last step, the chips become a specific biosensor by immobilization of selected capture molecules on the prepared substrates using a piezoelectric spotter (Nanoplotter 2.1, GeSim, Grosserkmannsdorf, Germany) with spot volumes of approximately 400 pl (8 nl each droplet, 20 droplets/spot, Figure 1b and Figure 2d).

### 2.2. Optical Biosensor Chip Reader Assembly

**Figure 3:**
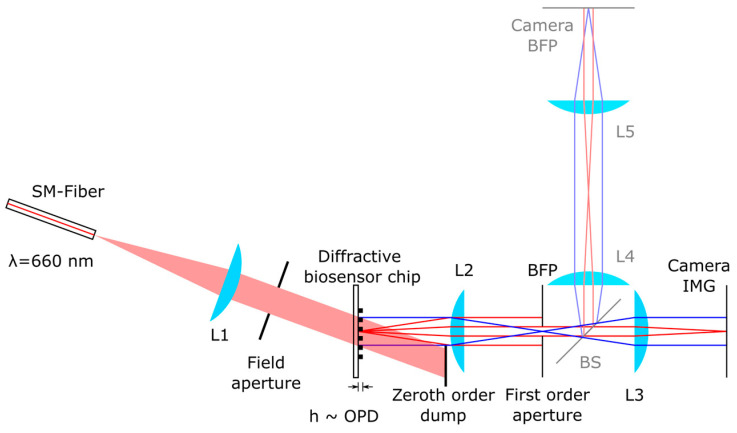
Schematic optical diagram of the imaging diffractometric biosensor reader including the diffractive biosensor chip. A single-mode fiber-coupled 660 nm Laser becomes collimated using L1 to illuminate the diffractive biosensor chip homogeneously. The illumination angle is tuned so that the first diffraction order leaves the biosensor chip in the normal direction. The grating-like biosensor chip allows the high-intensity zeroth-order diffraction to be separated from the first-order diffraction before illuminating any other optical component, efficiently avoiding scattering. The biosensor chip thus illuminated in the dark field is imaged by L2 and L3 on the camera IMG (blue lines: chief rays, red lines: marginal rays of the first diffraction order). A small aperture in the back focal plane (BFP) of L2 selects only the first diffraction order of the biosensor chip grating and suppresses other spatial frequencies. For technical diagnoses, a beam splitter BS is inserted, allowing for additional imaging of the BFP using L4, L5 and camera BFP.

For the biosensor chip readout, a corresponding dark-field imaging optics, selecting only the first diffractive order of the diffractive biosensor, is designed for this study (Figure 3). A single-mode fiber-coupled 660 nm Laser (51nanoL-S-660-30-Q02-P-5-2-18-0-150, Schäfter + Kirchhoff, Hamburg, Germany) with a reduced coherence length of ≈ 300 µm becomes collimated using L1 (AC254-100-A-ML, Thorlabs, Newton, NJ, USA) with a 100 mm focal length to illuminate the diffractive biosensor chip homogeneously. The illumination angle is tuned so that the first diffraction order leaves the biosensor chip in the normal direction. The grating period of the biosensor chip (a = 2 µm) allows for separating the first from the zeroth diffraction order using a filed aperture and a zeroth-order dump. This dark-field illumination avoids the >10,000 times more intense zeroth-order light becoming scattered by any other optical surface than the biosensor chip itself. The dark-field illuminated biosensor chip gets imaged by L2 (AC254-60-A-ML, Thorlabs, USA) with 60 mm focal length and L3 (C23-5028-5M-P, Basler, Ahrensburg, Germany) with a 50 mm focal length to camera IMG (acA3088-57um, Basler, Germany) with a monochrome 6 megapixel Sony IMX178 sensor. A small aperture with a typical diameter of d = 0.60 mm (NA = 0.0050) in the back focal plane (BFP) of L2 selects only spatial frequencies around the first diffraction order of the biosensor chip grating and suppresses other spatial frequencies. For technical diagnoses, a beam splitter BS (CCM1-BS013/M, Thorlabs, USA) is inserted, allowing for additional imaging of the BFP using L4 (AC254-50-A-ML, Thorlabs, USA) with a 50 mm focal length, L5 (C23-5028-5M-P, Basler, Germany) with a 50 mm focal length and the camera BFP (acA3088-57um, Basler, Germany). This additional optical branch allows for adjusting the first-order aperture but is not necessary for the reader principle. Note, the low numerical aperture of the imaging system being introduced by the first-order aperture intentionally leads to a low optical resolution of 7.5 lp/mm at the camera IMG since the structure of a single spot does not need to be resolved accurately. This enables the use of single spherical lenses instead of high-resolution imaging lenses.

The typical laser irradiance at the biosensor chip is 2 mW/cm² enabling for typical exposure times of camera IMG between 1 and 10 s. Images are taken linearly, without electronical gain and are saved as linear 16-bit tiff-files.

As a reference, for intensity normalization, a thin transmissive square-wave (black and white) amplitude grating made of gold with the same period and duty cycle as the biosensor is used (Figure 1a). The diffraction efficiency for the first diffracted order is $\eta_{SA}^{m=\pm 1}=1/\pi^{2}=10.13\%$ in paraxial approximation [29].

### 2.3. Quantitative Image Analysis

The raw images acquired with the reader assembly show the spatially-dependent local intensity of the first diffraction order of the dry biosensor chips. To obtain a quantitative readout of a biosensor chip, the raw images need to be converted in an analyte surface concentration which features grating-like spatial modulation. Since the absorption mean free path of biological analytes lies in the mm range for light with a wavelength of λ=660 nm, the biosensor can be modelled as a pure phase grating [30]. A suitable measure for a pure phase grating is the optical path length difference (OPD) Λ between land and pit:

(1) $$\Lambda =\frac{∆\varphi }{2\pi }\lambda$$

with ∆φ being the phase difference of light. The first-order diffraction efficiency $\eta_{SP}^{m=\pm 1}$ of a thin square-wave phase grating in paraxial approximation is given by [31]

(2) $$\eta_{SP}^{m=\pm 1}={\left(\frac{2}{\pi }\sin\frac{∆\varphi }{2}\right)}^{2}$$

It can be determined relative to the diffraction efficiency $\eta_{SA}^{m=\pm 1}$ of our reference which is a thin square-wave amplitude grating with equal geometry using the ratio of the corresponding first-order irradiances $I_{sen}$ and $I_{ref}$:

(3) $$\frac{\eta_{SP}^{m=\pm 1}}{\eta_{SA}^{m=\pm 1}}=\frac{I_{sen}}{I_{ref}}$$

Assuming homogeneous illumination, this ratio is free from reader specific losses. The irradiances on the camera caused by the first diffraction orders are given for the biosensor grating (phase only grating) with

(4) $$I_{sen}\left(x,y\right)=\frac{C_{sen}\left(x,y\right)-b_{sen}}{t_{sen}}$$

and for the reference grating (amplitude only grating) with

(5) $$I_{ref}=\frac{C_{ref}-b_{ref}}{t_{ref}}$$

where $C_{sen}\left(x,y\right)$ is the raw pixel value, $b_{sen}$ the dark pixel value, $t_{sen}$ the exposure time of the biosensor grating, $C_{ref}$ is the raw pixel value, $b_{ref}$ the dark pixel value, $t_{ref}$ the exposure time of the reference grating.

**Figure 4:**
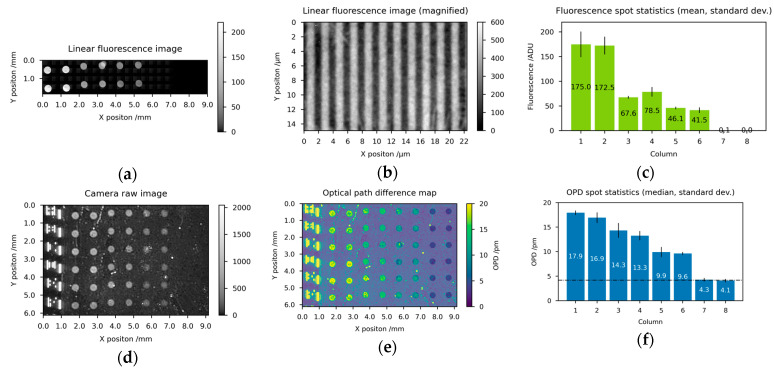
Fluorescence microscopy and diffractometric readout of a biosensor chip for short oligonucleotides with associated quantitative imaging analysis: (**a**) stitched linear fluorescence microscopy image (**b**) magnification of a spot detail of (**c**) mean and min-max of the 2 spots for each of the 8 columns of (**d**) linear raw image of camera IMG using 10 s exposure time and 2 mW/cm² irradiance, (**e**) normalized and OPD converted image from (**d**) with semi-transparent/white spot selection mask overlay, (**f**) median and standard deviation of the 6 spots for each of the 8 columns of (**b**). Columns 1–2 feature besides the two short single DNA strands two fluorescence labels for each successful hybridization event and therefore show the highest fluorescence value. Columns 3–6 feature a single fluorescence label for each successful hybridization and show a decreased fluorescence as well as a decreased OPD value compared to columns 1–2. The last two columns 7–8 could potentially contain residues of the wash buffer solutions and serve as a background measure.

**Figure 5:**
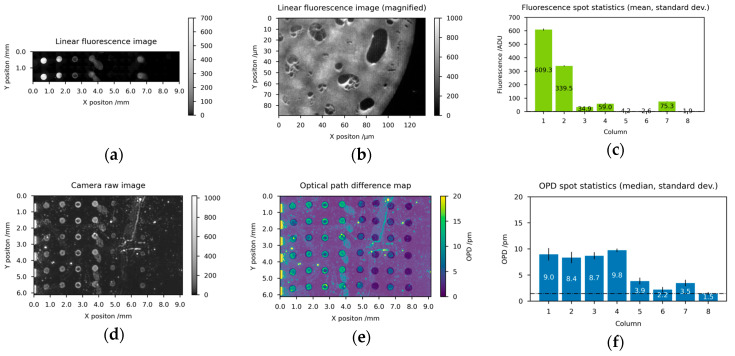
Fluorescence microscopy and diffractometric readout of a biosensor chip for proteins with associated quantitative imaging analysis: (**a**) stitched linear fluorescence microscopy image (**b**) magnification of a spot detail of column 1 in (**a**,**c**) mean and min-max of the 2 spots for each of the 8 columns of (**a**,**d**) linear raw image of camera IMG using 10 s exposure time and 2 mW/cm² irradiance (**e**) normalized and OPD converted image from (**d**) with semi-transparent/white spot selection mask overlay, (**f**) median and standard deviation of the 6 spots for each of the 8 columns of (**b**). Columns 1–3 are spotted with the nucleoprotein in decreasing concentration (300, 100, 20 µg/mL) to measure total anti-N antibodies binding events. Columns 4–6 are spotted with the spike protein (300, 100, 20 µg/mL) to measure total anti-S antibodies binding events. Column 7 contains the biotin monoclonal anti-human antibody (20 µg/mL) to serve as spotting control for both the OPD signal and the fluorescence signal (considering the background). The last column could potentially contain residues of the wash buffer solution and serve as a background measure.

**Figure 6:**
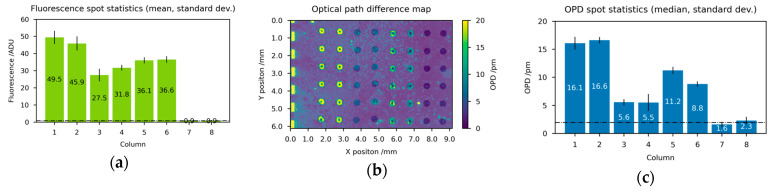
Fluorescence microscopy and diffractometric readout of a biosensor chip for protein-DNA interactions with associated quantitative imaging analysis: (**a**) mean and min-max of the 2 spots for each of the 8 columns of the linear fluorescence microscopy image (**b**) normalized and OPD converted image from the linear raw image of camera IMG with semi-transparent/white spot selection mask overlay, (**c**) median and standard deviation of the 6 spots for each of the 8 columns of (**b**). Columns 1–2 and 3–4 are spotted with modified oligonucleotides (N150B, 3′ end), featuring the high-affinity tag biotin at different concentrations (20 µmol/L, 10 µmol/L) and columns 5–6 are spotted with the same oligonucleotide but without a high-affinity tag (N150 20 µmol/L). Columns 7–8 are spotted with buffer only to provide a background measure.

For small phase differences where $I_{sen}\ll I_{ref}$ one gets the relation:

(6) $$\Lambda \left(x,y\right)\approx \frac{\lambda }{2\pi }\cdot \sqrt{\frac{I_{sen}\left(x,y\right)}{I_{ref}}}$$

which converts a raw image $C_{sen}\left(x,y\right)$ pixel wise into an image of OPD values $\Lambda \left(x,y\right)$. This calculation is applied in Figures 4–6 to convert the 12-bit linear raw IMG camera image shown in Figures 4d–6d into an optical path difference map displayed in Figures 4e–6e. For analyte detection only, the OPD is already a suitable measure. The OPD however does not only depend on the average height difference h between analyte and pure substrate (land and pit), but also on the refractive index of the analyte $n_{A}$ and the free space $n_{0}$:

(7) $$h=\frac{\Lambda }{n_{0}-n_{A}}$$

For most biological analytes one can assume $n_{A}=1.5$ [32], for dense DNA $n_{A}=1.53$ [33] and for the optical reader in air $n_{0}=1$. Using the mass density ρ of each individual analyte, the average height difference h can be converted into a mass density per unit area $\rho_{A}$ of an analyte at the gratings land:

(8) $$\rho_{A}=\rho \cdot h$$

For proteins the mass density is typically around $\rho =1.4\;g/{cm}^{3}$ [34] and for dense DNA $\rho =1.7\;g/{cm}^{3}$ [35]. 

Assuming a sensor grating with 100% mass density modulation, the total mass of analytes $m_{a}$ and capture molecules $m_{c}$ per spot calculates into

(9) $$m_{a}+m_{c}=\frac{\pi }{2}\cdot {\left(\frac{d}{2}\right)}^{2}\cdot h\cdot \rho$$

The spot diameter is given by d and the factor ½ comes from the fact that only half of the spot area is covered by grating land. 

In case the analyte is present in excess, causing saturation of the capture molecules, the number $n_{ca}$ of successful bindings or hybridizations per spot can be estimated with

(10) $$n_{ca}=\frac{m_{c}+m_{a}}{M_{c}+M_{a}}$$

The molar mass of the capture molecule is given by $M_{c}$ and of the analyte by $M_{a}$. For our study we observe 6 spots in a column of the same probe to give a statistical assessment of the reproducibility in detecting an analyte. The OPD of each spot is averaged and the median and standard deviation of the spots are calculated for each column.

### 2.4. Biochemistry Methods

Hybridization buffers (3X saline-sodium citrate (SSC) and 0.5% sodium dodecyl sulfate (SDS)), binding buffers (1X PBS, Tween-20 0.05%, Triton-X 0.125%), and washing solutions (PBS, SSC, SDS, ethanol) are purchased from Sigma-Aldrich (Madrid, Spain). 

For covalent binding of the capture molecules after spotting, the slide surfaces are exposed to 254 nm UV light (UV Bender NU-6KL, Wiesloch, Germany) for 10 min. Finally, unbound capture molecules are removed by washing with 0.1X saline sodium citrate (SSC)/0.5% sodium dodecyl sulfate (SDS) or 1X PBS respectively for 10 min at room temperature. Finally, the quartz substrates are dried under pressured air and stored at ambient temperature until use [27,28,36].

#### 2.4.1. Preparation of the Diffractive Biosensor Chip with Nucleic Acids

All nucleic acid capture molecules are dissolved in 1X Spotting Buffer 1 [31] to a final concentration ranging from 20 µmol/L to 0.5 µmol/L and spotted according to Figure 1b within the chip surface. Capture oligonucleotides (30 bases), which enable the detection of cytomegalovirus (CMV) [37] target DNA (Eurofins Genomics, Ebersbach, Germany), are designed to be fully complementary to the target molecules. Hybridization experiments are performed in a wet chamber at room temperature for 30 min. After target analyte exposure the substrates are washed successively for 2 min each in 2XSSC/0.1%SDS, 0.2X SSC, 0.1X SSC and 70% EtOH and rinsed with water. The sequences of the oligonucleotides used in this study are listed in Table 1.

#### 2.4.2. Preparation of the Diffractive Biosensor Chip with Proteins 

Purified proteins are dissolved in phosphate-buffered saline (PBS) to a final concentration ranging from 300 µg/µL to 5 µg/µL. N- and S-antigen preparations of SARS-CoV-2 (Institut Virion\Serion GmbH, Würzburg, Germany), are spotted according to Figure 1b onto the prepared chip surface as capture molecules. Next, surface blocking is done by adding a 10% BSA solution for 10 min. Biosensor chips are incubated with target solutions for 90 min (1:100 plasma sample) at 37 °C (Incucell, MMM Group, Munich, Germany). Plasma sample preparation is done according to Burgold-Voigt et al. [38]. Incubation is followed by several washing steps in 1X PBS, twice/5 min, 0.5% PBS/5 min and 70% EtOH/2 min. The final detection of human IgG antibodies is carried out with a secondary Goat anti-Human IgG (Invitrogen, Schwerte, Germany) antibody. The proteins used are reported in Table 2. Finally, the substrates are rinsed with water and dried under an air stream. 

#### 2.4.3. Validation Experiments and Characterization Techniques 

The hybridization and binding specificities are verified previously using ELISA and microarray [38] technologies. DNA molecules labeled with AlexaFluor^TM^488 or fluorescein isothiocyanate (FITC) fluorophore are purchased from Eurofins Genomics (Ebersbach, Germany). Antibodies labeled with AlexaFluor^TM^488 and goat anti-human antibodies are purchased from ThermoFisher Scientific (Schwerte, Germany), respectively.

Optical validation experiments are performed using fluorescence-labeled molecules and a fluorescence microscope. We use the inverse fluorescence microscope (ELYRA 7, Zeiss, Jena, Germany) equipped with: a HF Diode 488 nm, 495–590 nm BP Filter, a Zeiss EC Plan-Neofluar 10X/0.30 objective, and a 1.6X Tube-Lens and an upright fluorescence microscope (Axioskop 2, Zeiss, Jena, Germany) equipped with a collimated 470 nm LED (M470L4-C4, Thorlabs, USA), a 488 nm filter set (LF488-D-000, Semrock IDEX, Rochester, New York, NY USA), a Zeiss LD EC Epiplan-Neofluar 100X/0.75 HD DIC objective, and a Zeiss AxioCam MRm.

## 3. Results

### 3.1. Detection of Molecular Interactions between Oligonucleotides

As a first application scenario for the imaging diffractometric biosensor, we demonstrate the detection of short and labeled oligonucleotides (Figure 4) by hybridization between immobilized single-stranded capture DNA (N150_F, N150B, N150) and complementary target DNA (F150) from the cytomegalovirus (see Table 1). For this, the sensor chip is prepared with 8 columns of different capture molecules. Columns 1–2 are spotted with N150_F, columns 3–4 are spotted with N150B, and columns 5–6 are spotted with N150, each at 20 µmol/L dissolved in Spotting Buffer 1 [39]. These three single-stranded DNA types are nearly identical, but differently conjugated at the 3′ end and amino-modified at the 5′ end (see Table 1). For validation experiments, fluorescence labels are used which are small (~0.38 kg/mol) compared to the DNA in use. Columns 7–8 are spotted with buffer only to provide a background measure.

After preparation, the sensor chips are hybridized with the target DNA as described above. The readout of one representative biosensor chip as well as the associated quantitative imaging analysis is presented in Figure 4. Every two columns which are prepared under equal conditions show equal fluorescence (Figure 4a,c), as well as, OPD results (see Figure 4d–f and Equation (6)). Besides the two short single DNA strands, columns 1–2 feature two fluorescence labels for each successful hybridization event and therefore show the highest fluorescence value. Assuming a refractive index of $n_{A}=1.53$ and a mass density of $\rho =1.7\;g/{cm}^{3}$ for DNA, the background corrected OPD of 13.2 pm converts to 1.50 pg of successful hybridized DNA per spot (Figure 4f and Equation(9)). Columns 3–6 feature a single fluorescence label for each successful hybridization and show a decreased fluorescence (Figure 4c) as well as a decreased OPD value (Figure 4f) compared to columns 1–2. For the fluorescence (Figure 4c) a halving in intensity is to be expected since only half of the fluorescence labels should be present. However, the OPD signal should remain at a comparable level for columns 1–6 since the molar masses of all capture and target molecule pairs are approximately 17 kg/mol. The difference between the second and the third pair of columns (3–4 vs. 5–6) can be explained by a preparation-related local variation in the density of capture molecules leading to a drop in successful hybridized DNA to 1.09 pg (column 3–4) and 0.63 pg (column 5–6). Considering the number of fluorescence labels per hybridization, the results of the OPD and the fluorescence levels show a very good correlation. The last two columns could potentially contain residues of the wash buffer solutions and serve as a background measure (4.2 pm). The complete absence of fluorescence in columns 7–8 results in a perfect zero after dark current subtraction. 

### 3.2. Detection of Antibodies from Human Plasma against Nucleocapsid and Spike Proteins of SARS-CoV-2

As a second application scenario for the imaging diffractometric biosensor, we demonstrate the detection of antibody-antigen binding events and the potential to address current clinical applications. Serological samples, like blood plasma, are a challenge due to their complex composition, but clinical sampling is well established. As a clinically relevant example, we choose the detection of *n*- and S-antibodies against SARS-CoV-2 in human plasma samples. Immobilized nucleoproteins (SARS-CoV nucleoprotein) and spike proteins (SARS-CoV spikeprotein) on the biosensor chip (Table 2) allow for the specifical capture of N- and S-antibodies against SARS-CoV-2 in human plasma samples (1:100) (Figure 5). For validation experiments, the human antibodies are subsequently bound to an anti-human secondary antibody (1 µg/mL) (Table 2) with a fluorescence label. 

The sensor chip is again prepared with 8 columns of capture molecules in different concentrations in order to investigate the detection behavior. Columns 1–3 are spotted with the nucleoprotein in decreasing concentration (300, 100, 20 µg/mL) to measure total anti-N antibodies binding events. Columns 4–6 are spotted with the spike protein (300, 100, 20 µg/mL) to measure total anti-S antibodies binding events. Column 7 carries the biotin monoclonal anti-human antibody (20 µg/mL) to serve as spotting control for both the OPD signal and the fluorescence signal (considering the background). The last column could potentially contain the residues of the wash buffer solution and serve as a background measure. 

After preparation, the sensor chips are incubated directly with human plasma samples as described above. The readout of one representative biosensor chip as well as the associated quantitative imaging analysis is presented in Figure 5.

Fluorescence microscopy measurements (Figure 5a,c) reflect the nonlinearly decreasing concentration of capture nucleoprotein in columns 1–3. However, the background corrected OPD value remains at a constant value around 7.2 pm. A close inspection of column 1 and 2 spots, using fluorescence microscopy (Figure 5b), reveals a crowding effect [40] occurring due to the compact arrangement of the probe molecules on the surface. In this case, the required assumption of a well-modulated square wave strip grating molecule distribution for a diffractometric sensor is strongly violated and the sensor leaves its monotonous working range. Column 3 shows a rectangular grid apart from a drying ring and can be considered for OPD detection. So, only column 3, (of 1–3) with 20 µg/mL nucleoprotein, is suitably prepared for diffractometric readout. Comparing columns 3 and 7, the fourfold OPD signal reflects an efficient N-antibody binding to the immobilized nucleoproteins. However, the 50% fluorescence signal drop of column 3 compared to 7 shows a reduced binding of anti-human secondary antibodies with fluorescence labels (here necessary for the validation).

When analyzing columns 4–6, it is noticeable that only columns 4 and 5 show a fluorescence signal as well as an OPD signal above the background, whereas column 6 cannot be safely discriminated from the background (1.5 pm). The OPD signal level for columns 4–6 reflects linearly the decreasing mass density of bound antibodies on the chip surface. Due to the high signal-to-noise ratio, column 4 (of 4–6) with 300 µg/mL of spike protein is particularly suitable for diffractometric readout. Comparing fluorescence as well as OPD signals between columns 1–3 (SARS-CoV nucleoprotein) and 4–6 (SARS-CoV spikeprotein) a significant difference is obvious. Overall, these image analyses clearly show an increased level of anti-N antibodies compared to anti-S antibodies in this particular serological sample, this seems applicable for diagnostic testing [41]. The imaging diffractometric biosensor can thus be validated for addressing current clinical applications.

### 3.3. Simultaneous Detection of DNA and Protein Interactions

Related to the previous experiments, the imaging diffractometric biosensor is tested to detect proteins and DNA simultaneously (Figure 6). Columns 1–2 and 3–4 are spotted with modified oligonucleotides (N150B, 3′ end), featuring the high-affinity tag biotin (244 g/mol) at different concentrations (20 µmol/L, 10 µmol/L), and columns 5–6 are spotted with the same oligonucleotide but without a high-affinity tag (N150 20 µmol/L) dissolved in Spotting Buffer 1 [31] each. Columns 7–8 are spotted with buffer only, to provide a background measure.

After preparation, the sensor chips are incubated simultaneously with a complementary oligonucleotide featuring a fluorescence label (F150, 0.5 µmol/L) and a fluorescence-labeled anti-biotin antibody (anti-biotin IgG 20 ng/mL). 

The quantitative imaging analysis of one representative biosensor chip is shown in Figure 6. Each of the two columns which are prepared under equal conditions shows similar OPD as well as fluorescence results. Columns 1–2 (N150B, 20 µmol/L) show the highest signals (14.4 pm background corrected OPD) since they come with the highest spotting concentration and can feature a hybridized oligonucleotide and an additional antibody as a successful binding event. With half concentration, columns 3–4 show about half the fluorescence signal, but surprisingly only a quarter of the background corrected OPD signal (3.6 pm). Columns 5–6 (N150, 20 µmol/L) feature only a hybridized oligonucleotide without an anti-biotin antibody for each successful binding event and show a decreased background corrected OPD value (8 pm, 56%) and less fluorescence (76%) compared to columns 1–2. The last two columns could potentially contain remaining contributions from the wash buffer solutions and serve as a background measure (1.95 pm OPD).

The fluorescence signal is expected to be proportional to the number of labels and thus to the number of successful binding events; whereas the OPD signal is proportional to the total mass bound. The directional binding probability of proteins is lower due to their much higher molar mass (150 kg/mol) compared to the double-stranded DNA (dsDNA) fragment (17 kg/mol), but the molar mass for each successful binding is consequently increased. The increase in OPD of columns 1–2 in comparison to 5–6 can be easily explained by 9% of additional antibody bindings (in comparison to successful hybridizations). The simultaneous increase of fluorescence signal by 32% can be explained by 3.5 fluorophores on average being attached to a single antibody (2 to 8 fluorophores according to the manufacturer).

This third scenario (detecting proteins and DNA simultaneously) shows, that the imaging diffractometric biosensor sensor can be used to detect multi-molecular interaction. In addition, the sensor conceptually dispenses with additional labels.

### 3.4. Limit-of-Detection

Due to the chosen darkfield imaging approach, the optical limit of detection (LOD) for the presence of an analyte could be almost arbitrarily low. However, in the presence of unspecific bound sample components, particles, and surface roughness, the LOD is not defined by the optical readout but by the random refractivebackground which can also contribute to the intensity of the first-order diffraction of the biosensor grating. This contribution is linear in terms of OPD signal detection while it is not linear in terms of irradiance on the camera.

For our protocol, a typical OPD background is 2 pm with a standard deviation of 0.5 pm in the selected spatial frequency range. Assuming a refractive index for the analyte of $n_{A}=1.5$ and a mass density of $\rho =1.4\;g/{cm}^{3}$, this OPD can be converted in a background mass density per unit area of $\rho_{A}=5.6\pm 1.4\;pg/{mm}^{2}$ (Equations (7) and (8)). Taking three times the standard deviation of the background as LOD, results in $4.2\;pg/{mm}^{2}$, which is slightly lower than the value of $5\;pg/{mm}^{2}$ [42] obtained in a recent diffractometric non-imaging approach (spots had a diameter of d ≈ 400 µm). According to Brecht and Gauglitz [11], the most sensitive label-free optical transducer principles achieve a LOD of around $1\;pg/{mm}^{2}$ and rely on evanescent field techniques to monitor the affinity reaction via variation of refractive index by a change in ligand concentration. Note: The stated LOD values provide only an upper limit for the evaluation of the detection principle, as they are also dependent on experimental parameters such as preparation cleanliness. For our diffractometric sensor, we expect the LOD to decrease inversely proportional to the spot diameter, as the signal-to-noise ratio should increase linearly with the square root of the spot area and decrease due to a reduction in the measured spatial frequency band.

## 4. Discussion

In this work, we have developed an imaging diffractometric biosensor for the label-free detection of molecular interactions in biological and serological assays, suitable for the simultaneous detection of multiple types of target biomolecules. We demonstrate the label-free and sensitive detection of the following:

- DNA fragments from the human cytomegalovirus,
- Proteins namely the antibodies against SARS-CoV-2 in human plasma and
- DNA and protein interactions namely anti-biotin antibodies simultaneously.

The sensor is validated by means of fluorescence microscopy images. The observed correlation between the resulting optical path difference (OPD) values and the fluorescence signals supports the reliability and consistency of the biosensor assay in detecting the intended binding events. Since fluorescent labels are usually conjugated to the target molecules (such as antibodies or oligonucleotides) prior to incubation with the biosensor chip, the resulting fluorescence signal directly reflects the presence and extent of successful binding events. The OPD signal is influenced by the total mass bound, which may vary depending on the molecular weight and the number of bound molecules. The LOD is determined with an OPD value of 1.5 pm or a mass density per unit area of $4.2\;pg/{mm}^{2}$. The strengths/advantages of the reported biosensor can be summarized as follows:

- Label-free and simultaneous detection of biomolecules regardless of their chemical structure,
- Usage of universal pre-patterned surface functionalized chips independent of the application,
- Simple preparation of the pre-patterned functionalized chips to sensor chips for a specific application,
- Linear and absolute OPD response (assuming a well-modulated grating),
- Specificity given by the chemical molecule binding mechanism as in other assays that employ a single binding mechanism,
- High-temperature stability compared to refractometric optical biosensors (e.g., SPR and thin-film optical wave-guide biosensors) [43]
- Ability for live readout to study binding and unbinding kinetics [44],
- Ability to adjust the grating period according to specific needs and
- Simple and robust reader principles with low requirements on optics and camera.

The first strength, the absence of labeling, can also become a weakness of the sensor principle, since surface roughness, impurities, and unspecific bonding events can only be discriminated by their spatial frequency. However, spatial frequency discrimination does not necessarily achieve the same dynamic range as the chemical discrimination used in sensors based on fluorescence labels. To address this weakness, several strategies can be employed. One possibility is conducting the entire process of sensor chip preparation in a clean environment to mitigate the risk of contamination. A second possibility is to take advantage of the linearity of the OPD sensor principle and implement smart controls to detect and, if necessary, subtract signals of impurities. 

With this study, we extend the field of biosensors by the concept of a highly parallel imaging diffractometric biosensor. We propose an experimental setup for the visible wavelength range (660 nm) consisting of a diffractive biosensor chip and an associated optical reader assembly. The setup as such can be further developed or designed differently according to the application. Here, we only consider diffraction gratings caused by refraction differences of grating-like distributed biological molecules. If DNA or proteins are illuminated in the wavelength range of 280 nm or 260 nm, additional absorption properties can be utilized even without dyes. Whether it is possible to distinguish the sensor signals under UV illumination in DNA absorption more easily from impurities remains an interesting question. Unfortunately, LEDs in the 280 nm wavelength range are cheap but unsuitable for the spatial frequency filter due to their high spatial expansion. Spatially single-mode lasers for 266 nm, on the other hand, are expensive and the typical long coherence lengths can become an additional challenge due to reflections on surfaces. The adaptable grating period of the diffractive biosensor chip should not be too large, as small diffraction angles make dark-field illumination difficult and low-frequency irregularities of the substrate move into the passband of the spatial filter. Too small periods promote the crowding effect of capture molecules and analytes across the grating structure.

## 5. Conclusions

Our new imaging diffractometric biosensor has a clear parallelization advantage over single-spot diffractometric biosensors and simplifies assays compared to label-based approaches. It is based on a combination of dark-field illumination, spatial modulation of the analyte and band-limited imaging. We report an optical reader assembly that is very simple, stable, and robust, with low optical component requirements. Successful measurements and validation clearly demonstrate the functional sensor principle for multi-molecular interaction analysis. As the diffractive biosensor chips are suitable for the simultaneous detection of nucleic acid and protein interactions, they potentially enable applications for the diagnosis and therapy monitoring of diseases. It would also be conceivable to use the sensor principle for the parallel comparison of genotype and phenotype for pathogen detection.

## Figures and Tables

**Table 1:** List of capture and target DNA sequences for the detection of nucleic acids of the cytomegalovirus. (M: molar mass).

Name	Sequence	Modification		M [g/mol]
	(5′->3′)	5′	3′	
N150	TTTTTTCAGCATGTGCTCCTTGATTCTATG	AminoC6		9134.9
N150B	TTTTTTCAGCATGTGCTCCTTGATTCTATG	AminoC6	Biotin-TEG	9705.5
N150_F	TTTTTTCAGCATGTGCTCCTTGATTCTATG	FAM	AminoC6	9672.3
F150	CATAGAATCAAGGAGCACATGCTG	FITC		7962.8

**Table 2:** List of immobilized and investigated antibodies and proteins. (REF: reference number, LOT: batch number, c_0_: concentration of stock solution, M: molar mass).

Protein	REF	LOT	c_0_ [mg/mL]	Modification	M [kg/mol]
Biotin Monoclonal Antibody	53-9895-82	2143020	0.5	AlexaFluor488	150.0
Goat anti-Human IgG	A11013	2196582	2.0	AlexaFluor488	150.0
Ag-vs-sars-cov2-nucleoprotein	*n*-Protein	-	1840	-	47.0
Ag-vs-sars-cov2-spike-s1-s2	S-Protein	-	510	-	135.0

## Data Availability

Dataset available on request from the authors.
